# A longitudinal, population-based, record-linked natural experiment on the effects of extreme heat events on mental health in urban communities: a study protocol

**DOI:** 10.1136/bmjopen-2025-099771

**Published:** 2025-10-21

**Authors:** Emeka Chukwusa, Giulia Vivaldi, Lea Baecker, Emily Bowerman, Nick Bridge, Neil Davidson, Arturo de la Cruz, Antonio Gasparrini, Johanna Gibbons, Anne Guerry, Ryan Hammoud, Yingjie Li, Yougeng Lu, Lisa Mandle, David Osborn, Anders Rydstrom, Michael Smythe, Robert Stewart, Stefania Tognin, Matthew White, Justin Yang, Andrea Mechelli, Ioannis Bakolis

**Affiliations:** 1 Centre for Mental Health Policy & Evaluation, Department of Health Service & Population Research, Institute of Psychiatry, Psychology and Neuroscience, King’s College London, London, UK; 2 Department of Psychosis Studies, Institute of Psychiatry, Psychology and Neuroscience, King’s College London, London, UK; 3 J&L Gibbons, London, UK; 4 King’s College London, London, UK; 5 Environment & Health Modelling (EHM) Lab, Department of Public Health, Environments and Society, London School of Hygiene & Tropical Medicine, London, UK; 6 Natural Capital Project, Woods Institute for the Environment, Doerr School of Sustainability, Stanford University, Stanford, California, USA; 7 Department of Biology, Stanford University, Stanford, California, USA; 8 Department of Epidemiology and Applied Clinical Research, UCL, London, UK; 9 North London NHS Foundation Trust, London, UK; 10 Bethnal Green Nature Reserve, London, UK; 11 Department of Psychological Medicine, Institute of Psychiatry, Psychology and Neuroscience, King’s College London, London, UK; 12 South London and Maudsley NHS Foundation Trust, London, UK; 13 Herbert Smith Freehills LLP, London, UK

**Keywords:** MENTAL HEALTH, EPIDEMIOLOGY, Climate Change, Electronic Health Records, Observational Study

## Abstract

**Abstract:**

**Introduction:**

Studies suggest that extreme heat events can have negative effects on mental health. However, characterisation of these effects in urban communities remains limited, and few studies have investigated the potential modifying effects of demographic, clinical and environmental characteristics. The aim of this study is to address this knowledge gap and quantify the impacts of extreme heat on mental health, health service use and mental well-being in vulnerable urban populations.

**Methods and analysis:**

In this multidisciplinary project, we will assess mental health outcomes in different populations by bringing together two distinct datasets: electronic health record (EHR) data on mental health service users and data from general public participants of Urban Mind, a citizen science project. We will use EHRs from the South London and Maudsley NHS Foundation Trust (SLaM) and the North London NHS Foundation Trust (NLFT), from six boroughs which collectively cover more than 1.8 million residents in Greater London, to capture mental health service use and mortality among people with existing diagnoses of mental illness across 2008–2023. We will use smartphone-based ecological momentary assessment data from Urban Mind to measure mental well-being in the general population (2018–2023). These datasets will be linked to high-resolution spatiotemporal data on temperature, fine and coarse particulate matter (PM_2.5_, PM_10_), nitrogen dioxide (NO_2_), Normalised Difference Vegetation Index (NDVI) and density of large mature tree canopy. We will employ novel quasi-experimental designs, including case time series and case-crossover analysis, to examine the impact of extreme heat on mental health and explore effect modification by sociodemographic, clinical and environmental factors, including air pollution and types of green space coverage. We will also develop a microsimulation model combined with the InVEST urban cooling model to assess and forecast the mental health and social care impacts of extreme heat events and the mitigation of these impacts by different green space coverage and pollution-reduction policies. With a core team composed of researchers, community organisations, industry partners and specialist policy experts, this project will consider lived experience, benefit from broad stakeholder engagement and address gaps in policy and practice.

**Ethics and dissemination:**

Each component of this project has been approved by the relevant ethics committee (ref RESCM-22/23-6905 for Urban Mind, LRS/DP-23/24-41409 for the co-development of a screening tool, 23/SC/0257 for the SLaM EHRs, and 24/EE/0178 for the NLFT EHRs). Our dissemination plan includes peer-reviewed scientific articles, policy briefs, a practical guide on fostering ecological and human resilience at the neighbourhood level, and a technical guide for planting and improving the growing conditions of large canopy trees.

**STRENGTHS AND LIMITATIONS OF THIS STUDY**

- This natural experiment leverages data from distinct datasets that capture mental health outcomes in different highly diverse populations, allowing characterisation of the impact of extreme heat events in people with and without an existing diagnosis of mental illness and considering numerous potential individual-level and area-level confounders.
- Linkage of electronic health records and smartphone data with state-of-the-art high-resolution spatiotemporal datasets of environmental factors will enable a detailed characterisation of the impact of extreme heat and the potential modifying effects of air pollution and exposure to green space.
- The inclusion of academics, clinicians, community organisations, industry partners and specialist policy experts within our core team offers the advantage of improved research design, broader stakeholder engagement and enhanced translation of knowledge into policy and practice.
- The mental health data used face various challenges: assessment of mental well-being in a self-selected cohort, which relies on access to a smartphone app, may introduce selection bias and prevent generalisation of our findings to the wider population, whereas analyses of routinely collected health data can struggle with poor data quality and limited information on potential confounders.
- Although our findings could generalise to cities in other high-income countries, the generalisability to low-income and middle-income country urban settings, which often have much higher temperatures and pollution levels and lower access to green spaces and psychiatric healthcare provision, will remain uncertain.

## Introduction

 Many countries around the world are experiencing more frequent and intense heatwaves.^1^ This effect can be concentrated in cities owing to the urban heat island effect, where the combination of increased heat output, heat-absorbent surfaces and low levels of vegetation lead to higher land-surface and air temperatures and reduced heat dissipation.^2 3^ Cities are already experiencing record temperatures,^4^ and with urban populations predicted to double by 2050,^5^ the number of people exposed to extreme urban heat will grow substantially over the coming decades.

The negative impacts of extreme heat events on physical health are long recognised,^6^ with policies in place to support the physical well-being of the general population and vulnerable groups.^7^ A more recent finding is that extreme temperatures can also have profound negative effects on mental health, but this is under-researched—fewer than 1% of 54 000 papers on climate change published before 2020 mentioned mental health.^8^ Two recent systematic reviews have linked higher temperatures with psychiatric hospital attendance for mental health problems and increases in suicide incidence and risk.^9 10^

Poor mental health is a global health concern.^11^ Mental disorders and subjective well-being are associated with worse general health outcomes and reduced quality of life.^12^,^14^ Beyond the individual cost, poor mental health has a large societal burden, being estimated to cost the UK £300 billion per year in economic, human and health and care costs.^15^

As temperatures and urban populations increase, characterising the effect of extreme heat on mental health is crucial to be able to plan for and mitigate its broader impact on individuals and society. To do so effectively, we need to consider how people’s environments affect their exposure and response to extreme heat and how we can design environments to improve thermal comfort and overall well-being. However, few existing studies have focused on people who live in dense urban areas,^16 17^ despite the well-established finding that urban living is associated with higher rates of mental health issues.^18^ Additionally, most studies to date have considered suicide or hospital attendance for mental health problems,^9 10^ with few considering mental well-being in the general population.^19^,^22^ Furthermore, disparities by sociodemographic and clinical characteristics have been investigated for few mental health outcomes,^20^ and no studies, to our knowledge, have considered mitigation of the mental health and social care impact of extreme heat^23^ by different types of urban greening^24^ and pollution-reduction policies.^25^

Heat exposure can vary by sociodemographic factors owing to neighbourhood characteristics, with older and deprived populations and minoritised ethnicities experiencing increased exposure to heat and being more vulnerable to heat stress.^21 22^ These higher levels of heat exposure are of particular concern for population subgroups more likely to be negatively impacted by extreme heat, such as older adults,^10^ people with existing mental health disorders^26^ and people of lower socioeconomic status.^20^ There is also evidence that long-term exposure to poor air quality is linked to poor mental health outcomes, leading to increased risk of depression,^27^ anxiety,^28^ cognitive decline,^29^ mental health service use,^30 31^ multiple long-term conditions^32^ and oxidative stress,^33^ with some studies suggesting an interactive effect of heat and air pollution on physical^34^ and mental health.^35^ Additionally, green space—which can reduce heat intensity and boost mental well-being^36^—may modify the effect of extreme heat on mental health, with a previous study showing that exposure to greater tree canopy cover may mitigate the effects of extreme heat exposure on mental and behavioural disorders.^26^

Finally, combining results from existing studies is complicated by the wide range of definitions used for extreme heat in the existing literature, mixing between objective and relative temperature thresholds and single-day and multiday durations.^9^ These inconsistencies make it difficult to quantify the overall impact of extreme heat, even within the same geographical area.

## Aims

**Figure 1:**
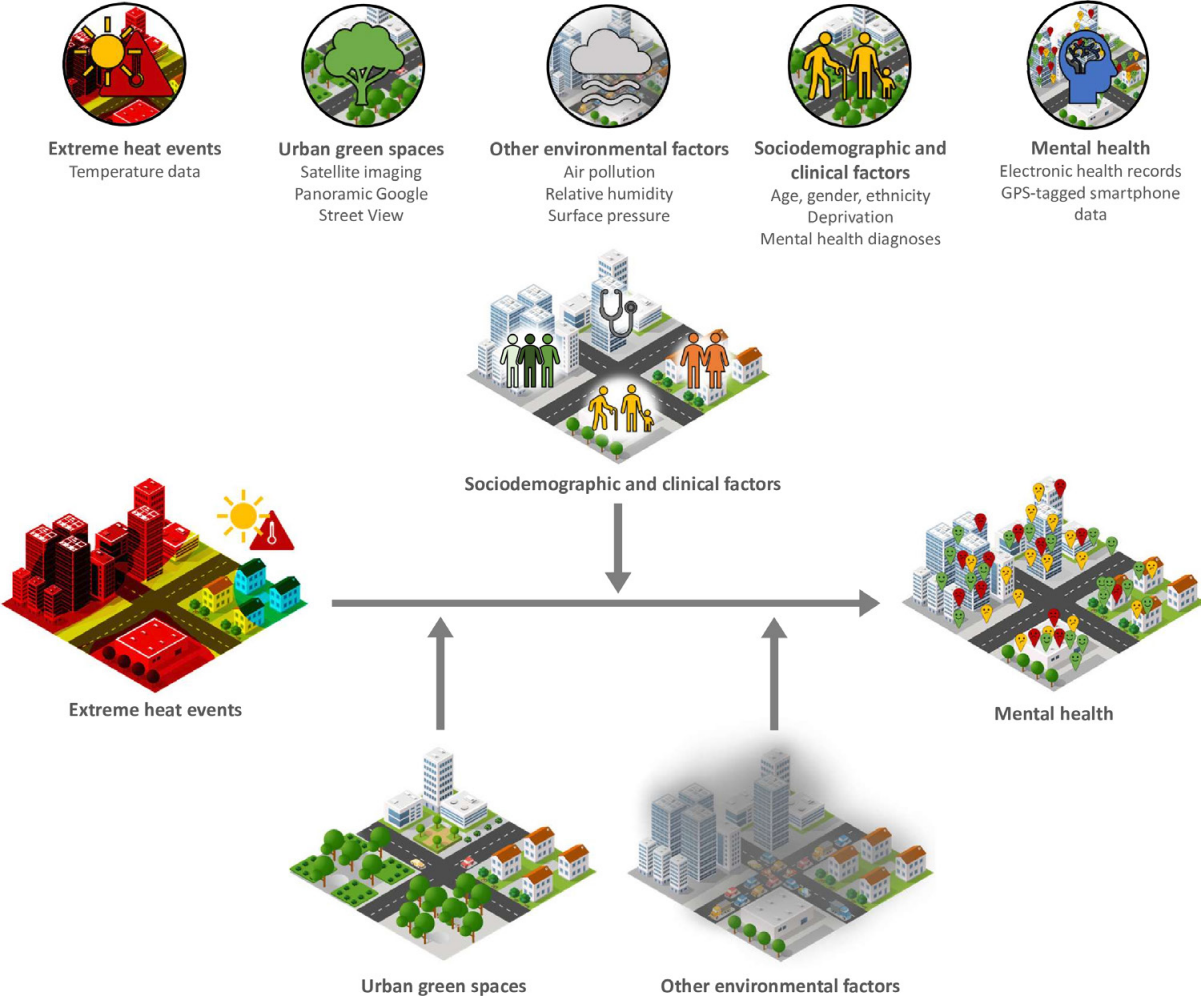
Data types and expected relationships. GPS, global positioning system.

Our primary aim is to address this knowledge gap and quantify the impacts of extreme heat on mental health, health service use and mental well-being in vulnerable urban populations. To do so, we will bring together distinct datasets that capture mental health outcomes in different populations, unifying our findings by using consistent definitions of extreme heat and temporal overlap of the datasets. By linking these datasets with high-resolution spatiotemporal datasets of environmental characteristics, we will explore the modifying effect of key sociodemographic and environmental factors (figure 1).

Our secondary aim is to support translation of our findings into policy and practice. To enable this, we will assemble a team of multidisciplinary and multiprofessional expertise comprising academics, community organisations, industry partners and specialist policy experts. We will extend our research into the community, involving people with lived experience of mental illness, mental health professionals and urban residents to improve understanding, develop clinical screening tools and produce guides for local communities on mitigating and adapting to the impacts of climate change. We will provide relevant evidence and analysis to improve policy and regulatory interventions at national, regional and local authority level—for example, in promoting mature tree canopies in urban settings, the design of green space and infrastructure, and wider delivery of net zero and environmental goals.

## Objectives

**Figure 2:**
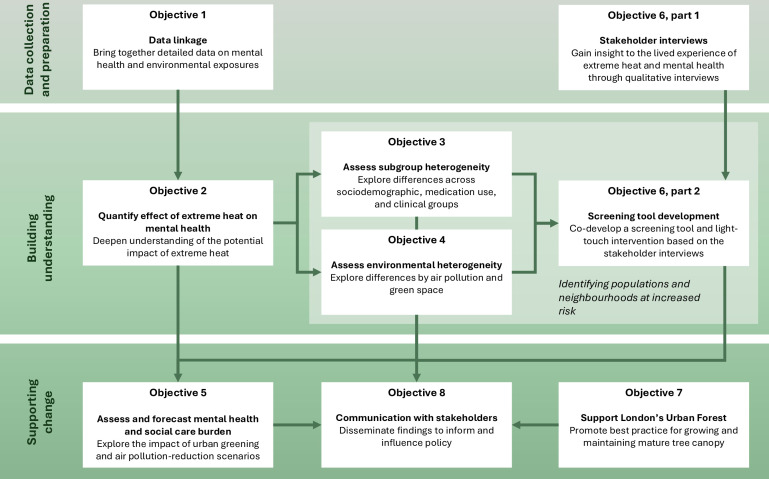
Overview of the project and connections between its objectives.

We have eight key objectives (figure 2).

1. Create data linkages between mental health data and environmental datasets.
  - Use the Clinical Records Interactive Search (CRIS) application to extract data on mortality, emergency hospital admissions and mental health service use among South London and Maudsley NHS Foundation Trust (SLaM) and North London NHS Foundation Trust (NLFT) service users and link these records to temperature, air pollution and green space data according to area of residence.
  - Link smartphone data from the Urban Mind app to temperature, air pollution and green space data using geographical coordinates.
2. Quantify the effect of extreme heat on mental health, looking separately at mental health service use, emergency hospital admissions and mortality within the CRIS datasets and mental well-being within Urban Mind.
3. Assess the potential heterogeneity of the effect of extreme heat on mental health across different sociodemographic, medication use and mental health diagnostic groups.
4. Assess the potential heterogeneity of the effect of extreme heat on mental health according to environmental features such as particulate matter, NO_2_ and various elements of green space exposure.
5. Assess and forecast the mental health and social care burden that could be attributable to extreme heat with the use of a microsimulation model, in which we will derive effect estimates of extreme heat on mental health in subgroups of interest, and then compare these effect estimates across different urban greening and air pollution-reduction scenarios.
6. Gain insight into the lived experience of extreme heat and mental health, by (A) conducting qualitative interviews with key stakeholders (including people with a history of mental illness and mental health professionals) to gain insight to the experience of extreme heat in vulnerable populations and (B) through codevelopment of a screening tool and light-touch intervention to enable clinicians to identify the most vulnerable service users and provide recommendations.
7. Identify opportunities to employ best practices for growing mature large canopy trees in London while visually communicating project outputs to policy-makers and broader interest groups such as built environment practitioners, developers, local authorities and the community.
8. Draw the attention of policy-makers to the impact of extreme heat on mental health and influence their development or revision of relevant policy and regulatory analysis and frameworks, using various means including published material, media and presentations.

## Methods

### Study design

We will conduct a natural experiment to evaluate the association between extreme heat and mental health outcomes. Retrospective cohorts will be constructed using two different types of data: anonymised electronic health record (EHR) data from SLaM and NLFT and smartphone-based ecological momentary assessment (EMA) data from the Urban Mind dataset.

### Data sources

#### Urban Mind

Urban Mind is a smartphone-based citizen science project on the impacts of the urban environment on mental health (http://www.urbanmind.info). This EMA app was developed as part of the Urban Mind research project—a collaboration between King’s College London, landscape architects J&L Gibbons and arts foundation Nomad Projects—which aims to inform future urban planning and social policy to improve design and health in urban environments. By relying on the repeated sampling approach of EMAs, the app is designed to provide insight into dynamic changes in mental states while maximising ecological validity by collecting data in real-world environments.^37^ The app has been in use across various research projects since 2015,^37^ with the current version launched in May 2018. Participation in the project is self-selected and anonymous and has been encouraged through the project website and social media platforms.^37^ The dataset includes more than 56 000 assessments from over 5600 people globally, with collection ongoing. Approximately one-third of the Urban Mind cohort has a diagnosis of mental illness. The Urban Mind app is available for both Apple iPhone and Android devices and is free to use. Additional details about the Urban Mind project and the app can be found in previous publications.^37 38^

Participation in Urban Mind consists of an initial baseline assessment and a maximum of 42 EMAs per participant, spread out over 14 days. The baseline assessment comprises data on demographics, socioeconomic factors, sleep patterns and self-reported mental health history. After the baseline assessment, the app schedules three EMAs per day over the subsequent 14 days. Based on each participant’s self-reported sleep schedule, waking hours are divided into three windows, with one assessment scheduled randomly in each window. Once notified that an assessment is ready, participants are given 1 hour to respond to any given assessment before it is marked as incomplete.

The EMAs capture information about participants’ perceptions of their surrounding built environment, the surrounding social environment and an individual’s mental well-being and activities. Locations of each assessment are geotagged with coordinate locations represented as longitude and latitude.

**Figure 3:**
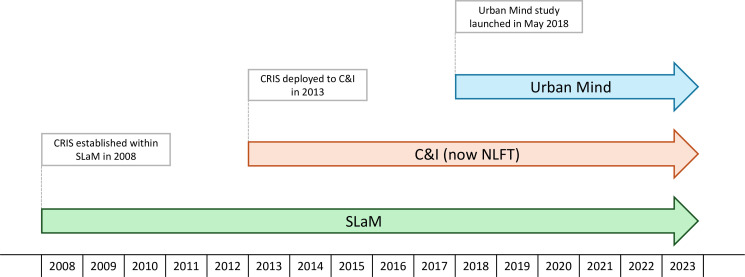
Timeline of mental health datasets. C&I, Camden and Islington NHS Foundation Trust; CRIS, Clinical Records Interactive Search; NLFT, North London NHS Foundation Trust; SLaM, South London and Maudsley NHS Foundation Trust.

Our Urban Mind cohort will include all participants aged 16 years or older with observations in Greater London, from study launch in 2018 until 2023 (figure 3).

#### SLaM and NLFT CRIS

SLaM is one of the largest providers of secondary mental healthcare in Europe,^39^ providing mental healthcare for service users in four London boroughs (Lambeth, Croydon, Lewisham and Southwark), which range from inner urban to suburban settings. The catchment area served by SLaM comprises approximately 1.36 million residents^30^ and is ethnically diverse, with a substantially higher proportion of people from minority ethnic groups compared with the rest of England.^40^ In terms of age, gender and socioeconomic status, the SLaM catchment does not differ substantially from London as a whole.^39^ After housing costs, average poverty levels in the four boroughs are largely similar to the London average and significantly higher in Lewisham than the England average.^41^ However, health and social inequalities are high, with both Croydon and Southwark home to areas in the highest and lowest decile of the Index of Multiple Deprivation (IMD).

The NLFT is a large mental health provider serving the London boroughs of Barnet, Camden, Enfield, Haringey and Islington. It was formed in November 2024, from the merging of the Camden and Islington NHS Foundation Trust (C&I) with the Barnet, Enfield and Haringey Mental Health NHS Trust. At the time of writing, data availability from the NLFT is restricted to records from C&I, which served approximately 470 000 residents in the London boroughs of Camden and Islington.^42^ This catchment area has a similar population composition to other boroughs in London, but with a higher proportion of younger people compared with the rest of London and England as a whole. The two boroughs differ substantially in their socioeconomic status, with Camden having the highest poverty rate after housing costs in London, at twice the level of Islington, whose rate is significantly lower than the London average.^41^ As with the SLaM boroughs, inequality in Camden and Islington is high.

Both SLaM and the NLFT operate a fully EHR platform known as the CRIS system. Established within SLaM in 2008 and deployed to C&I (now NLFT) in 2013, this platform allows access to anonymised individual-level records of mental health service users for both NHS Foundation Trusts.^42^

CRIS contains a comprehensive record of all clinical information recorded throughout patients’ journeys within Trust services. This includes patients’ demographic data (eg, gender, self-assigned ethnicity), details of referrals and transfers, clinical assessments, care plans and medication, and clinical activity and reviews, from both structured and unstructured free text sources (eg, routine case notes, correspondence and medication notes).^40 42^ CRIS also includes details of mental health outcomes such as the Mini-Mental State Examination and Health of the Nation Outcome Scales (HoNOS).^39^

Access to both databases is overseen by respective oversight committees and only authorised researchers are provided access to deidentified patient records. Studies using data from the SLaM and NLFT databases receive ethical approval from the Oxford Research Ethics Committee and NHS REC East of England—Cambridge Central, respectively.^40 42^ As of October 2024, the NLFT CRIS for Camden and Islington is estimated to contain approximately 180 000 patient records, whereas SLaM CRIS is estimated to contain around 500 000 patient records.

Our CRIS cohort will thus include residents of the six London boroughs aged 3 years or older (within SLaM) or aged 18 years or older (within Camden and Islington) who had contact with SLaM from 2008 to 2023 or C&I (now NLFT) from 2013 to 2023 (figure 3).

### Outcomes

#### Mental well-being score

In Urban Mind, mental health is assessed with 10 items of momentary mental well-being, composed of five positive affect items (feeling confident, relaxed, happy, connected with other people or energetic) and five negative affect items (feeling anxious, stressed, down, lonely or tired). For each of these 10 items, participants are asked to indicate their level of agreement with statements of the form ‘Right now I feel…’, using a 5-point Likert scale ranging from ‘strongly disagree’ (1) to ‘strongly agree’ (5). An overall mental well-being score is then defined as the sum of the positive affect items and the reverse of the negative affect items, giving a total score ranging from 10 (low mental well-being) to 50 (high mental well-being). Participants are additionally asked to rate their overall mental well-being from ‘very poor’ to ‘very good’, with an option for ‘not sure’. The psychometric properties (ie, validity and reliability of the well-being score) will be checked while taking into account the hierarchical nature of the dataset.

#### Mental health service use

In SLaM and NLFT, primary and secondary diagnoses are recorded by clinicians responsible for patient care using the International Classification of Diseases, 10th Revision (ICD-10) codes (table 1). We will focus on primary conditions recorded in the structured fields of CRIS, supplemented by those recorded in the unstructured fields. The latter will be extracted using the General Architecture for Text Engineering, a facility within the CRIS environment used to extract unstructured clinical notes or free text.^40^

**Table 1:** List of ICD-10 codes used in previous studies assessing mental health service use and environmental exposures

Conditions	Clinical codes	Reference
Organic, including symptomatic, mental disorders	F00–F09	^31 69^
Mental and behavioural disorders due to psychoactive substance use	F10–F19	^69^
Schizophrenia, schizotypal and delusional disorders	F20–F29	^30 69^
Mood (affective) disorders	F30–F39	^30 69^
Neurotic disorders	F40–F59	^69^
Disorders of adult personality and behaviour	F60–F69	^69^
Mental retardation	F70–F79	^69^
Developmental disorders	F80–F98	^69^

ICD-10, International Classification of Diseases, 10th Revision.

Mental health service use will be measured using CRIS records on care received by service users. We will include the number of hospital admissions, length of inpatient stay, number of emergency department visits, community mental health team (CMHT) events, admissions for self-harm, suicide and crisis episodes. Inpatient days will be calculated as the difference between dates of admissions and discharge from inpatient wards. CMHT events will include daily counts of face-to-face or virtual appointments with CMHTs (including Home Treatment Teams and emergency contacts), who provide community-based services for people with mental health within SLaM and the NLFT. Daily admissions for self-harm and suicide will be identified using diagnostic ICD-10 codes from previous studies.^43 44^ Crisis episodes will be defined as counts or frequency of detention under the Mental Health Act (sections 2, 3, 135 and 136).

### Extreme heat exposure

To capture different elements of extreme heat exposure, we will consider acute extreme heat events (ie, high maximum daily temperatures, regardless of their duration) and sustained extreme heat events (ie, heatwaves, where temperatures remain elevated over time).

We will explore different thresholds for acute extreme heat events. First, we will use the UK Health Security Agency decision-making aid temperature thresholds, which are based on a maximum daytime temperature (≥28.0°C for London and ≥27.0°C for the rest of the UK).^45^ Second, we will use relative thresholds, defined as maximum daytime temperature ≥95th percentile of the location-specific summer temperature distribution (comprising June, July and August), consistent with various previous studies.^10^ We will also conduct exploratory analyses to identify specific minimum thresholds in our data, and whether these differ according to outcome. To explore the impact of sustained heat exposure, we will define heatwaves as a period of at least three consecutive days of extreme heat,^46^ with the thresholds defined above.

Temperature data used to define these outcomes will be extracted from HadUK-Grid, a dataset of gridded climate observations for the UK developed by the UK Met Office.^47^ We will extract daily maximum and minimum values of air temperature on a 1×1 km grid.

### Environmental covariates

#### Air pollution

Air pollution will be measured using a satellite-based spatiotemporal machine learning model,^48^ which estimates daily levels of PM_2.5_, PM_10_ and NO_2_ at a 1 km² resolution across large spatial domains. Five machine learning algorithms are applied in multiple stages to integrate spatial and spatiotemporal variables to train a prediction model, which is then used to estimate daily concentrations comprehensively across the spatial domain. Air pollution data will be mapped to observations as described above.

#### Meteorological variables

Total daily precipitation (mm) will be extracted from HadUK-Grid.^47^ Daily surface pressure, measured in pascals, will be obtained from the ERA5-land reanalysis dataset,^49^ which provides estimates at a resolution of approximately 9 km². Relative humidity (in %) will be calculated via water vapour pressures obtained from applying the August-Roche-Magnus equation to temperature and dewpoint temperature data extracted from ERA5-land.^49^

#### Green space exposures

Exposure to urban green space will be assessed using satellite imagery, Google Street View (GSV) and Land Use Land Cover Maps, with different strategies developed to assess urban green space exposure for Urban Mind data and EHR data.

For Urban Mind, in which observations are linked with coordinate locations, panoramic GSV images will be accessed based on participants’ coordinates for assessments where participants have stated they were outdoors. For assessments marked as indoors, and where GSV images are not available, we will use 10 m resolution multispectral satellite images from the freely available Sentinel 2 satellite programmes to extract the NDVI as an alternative indicator for green space exposure within a 300 m search radius.

For the CRIS datasets, which are provided at the postcode or Lower Super Output Area (LSOA) level, we will calculate the four seasonal NDVI scores every year within a 300 m radius of each residential location, using 30 m resolution Landsat imagery during our study period from 2008 to 2023. Additionally, land use, land cover and tree canopy data will be combined to estimate the degree of mature street tree canopy at the neighbourhood level.^50^ Finally, we will assess accessibility to the seven park categories defined in The London Plan, comprising regional parks, metropolitan parks, district parks, local parks and open spaces, small open spaces, pocket parks and linear open spaces. This will be achieved using a Gaussian distance decay weighting function to estimate park access within a predefined distance radius of each residential address, based on recommendations on green space access from The London Plan.^51^

The link between green space exposure and heat assessment will then be adapted to run the InVEST Urban Cooling Model (UCM), developed by the Natural Capital Project at Stanford University. This model is part of a widely used toolkit for assessing ecosystem services.^52^ The UCM estimates heat mitigation by incorporating the effects of shade, evapotranspiration, albedo and proximity to cooling features such as parks. It further translates land-use and land-cover patterns or scenarios into projected changes in urban heat island intensity, enabling evaluation of how green infrastructure contributes to reducing heat stress and supporting human health. This model will be applied across various climate and land-use scenarios to evaluate the benefits of green space on mental health outcomes.

### Dataset-specific covariates

The selection of individual-level and area-level covariates will be informed by previous studies based on factors known to influence exposure to extreme heat and mental health outcomes or use of mental health services. We will include individual-level variables such as age, sex or gender, ethnicity, marital status, counts of mental and physical health comorbidities, and other individual-level clinical characteristics recorded within Urban Mind and CRIS.

#### Urban Mind

We will include participant information recorded in the baseline assessment, such as age, gender, ethnicity, highest educational achievement, occupation and history of mental health conditions.

We will additionally include each participant’s self-reported contact with nature. In EMAs, participants are asked whether they can see (or, where appropriate, hear) four natural features: trees, plants, birds and water. Responses to these four questions will be used to assess each participant’s momentary and historical contact with different elements of nature and can be combined to produce a nature diversity score, as previously described.^38^

#### Clinical Records Interactive Search

At the individual level, we will include age, sex and ethnicity. At the area level, we will include population density, socioeconomic status and social fragmentation. Population density will be measured at the LSOA level using the 2021 census estimate. LSOAs are small geographical units with an average population of 1500 people. Area-level socioeconomic status will be derived from the IMD. The IMD is a composite measured at the level of LSOAs, comprising seven domains: income, employment, health deprivations, disability, education, skills and training, and barriers to housing and service. IMD will be grouped into quintiles, ranging from the most deprived (1) to the least deprived (5). Social fragmentation at the LSOA level will be derived using a similar approach used in a previous study^53^ comprising a composite score of four indicators: the number of single people, those renting privately, population turnover and one-person household. We will include other measures collected in routine clinical practice such as the HoNOS and active days within SLaM or the NLFT. HoNOS are 4-point measures of health and social functioning rated from 0 to 4 (a score of 0 indicates that the problem is least serious and 4 the most).^54^ Active days is a measure of the time a patient is in contact with CRIS, which will be used to quantify patient follow-up time.^31^

### Statistical methods

#### Data linkage (objective 1)

We will link the Urban Mind and CRIS datasets with the environmental covariates described above. For Urban Mind, environmental covariates will be mapped to participants’ EMAs by date of observation and the longitude and latitude recorded for each assessment. For CRIS analyses, environmental covariates and service use data will be matched based on service users’ residential LSOA or postcode address data and date of contact with mental health services.

#### Descriptive statistics

Individual-level, area-level and environmental exposures will be described using descriptive statistics. Continuous variables will be described using the mean and SD or median and IQR, as appropriate. Categorical variables will be described using proportions or percentages. We will create scatter plots of exposures and outcome variables against time to visualise systematic patterns, including seasonal and long-term trends. For Urban Mind, we will use relevant cartographic methods to explore the relationship between data from Urban Mind, deprivation indices and tree cover to identify opportunities for real-world interventions to improve London’s Urban Forest. Landscape analysis,^55^ the method by which landscape architects assess landscape conditions, will be employed to observe, analyse and identify opportunities for prototype tree planting sites.

#### Analysis of the association between extreme heat and mental well-being (objective 2)

To model associations between extreme heat and mental well-being within Urban Mind, we will consider two modelling approaches. First, we will use multilevel mixed models, with random intercepts for participants. We will begin by testing for a linear trend, including temperature as a continuous outcome. To assess the impact of acute extreme heat events, we will construct multilevel logistic regression models with exposure to extreme heat as a binary outcome. As extreme heat may have a delayed effect on mental health outcomes, we will include lags to explore the longer-term effects of extreme heat exposure, depending on data availability, with the potential to impute missing data. We will assess whether non-linear forms better describe the relationship between temperature and well-being score; if this is the case, we will consider the use of the case time-series approach.^56^ This approach offers a flexible, self-matched method suited to the analysis of acute outcomes associated with time-varying exposures and allows complex exposure-lag-response relationships.

#### Analysis of the association between extreme heat and mental health service use (objective 2)

Within CRIS, we will use a time-stratified case-crossover approach to examine the effect of extreme heat on mental health outcomes—an approach that has been widely used to examine the effect of transient exposures on health outcomes.^57^ In this type of analysis, each case acts as their own control, with follow-up split into case days and control days. Case days will be defined as the dates of mental health event or use (eg, hospital admissions, emergency department visits) whereas control days are days with no recorded event. Case and control days are matched by splitting the time-series data into equally sized, non-overlapping strata defined by the same day of the week, year and month.^58^ As our outcomes of interest are counts, we will use a conditional quasi-Poisson regression model for overdispersed count, combined with a distributed lag non-linear model^59^ to estimate the associations between short-term exposure to extreme heat and mental health use.

We will create matched cohorts within CRIS, matching participants exposed to extreme heat events during follow-up to participants who remained unexposed. Our initial approach to constructing the unexposed cohort will be propensity score matching. We will use logistic regression to derive a propensity score for each participant, which will represent each participant’s probability of being exposed to an extreme heat event. In this model, the outcome will be exposure to an extreme heat event, and the dependent variables will be sociodemographic variables that best predict the outcome. We will use these propensity scores to match exposed participants to unexposed participants, ensuring that the distribution of propensity scores is as similar as possible between the exposed and unexposed groups.

We will reanalyse the association using aggregate-level small area geographical units such as LSOAs. We will adopt a Bayesian hierarchical modelling framework to account for both spatial structured and unstructured random effects with adjustment for relevant confounders. The model will be parameterised using the Besag-York-Mollié conditional autoregressive prior distribution. Small area variations in the risk of outcomes will be presented as the posterior means accompanied by 95% credible intervals. The estimates will be mapped and visualised using Choropleth maps to show variations.

#### Assessment of the heterogeneity of the effect of extreme heat by mental health outcomes and population subgroups (objective 3)

We will construct separate models to assess potential heterogeneities of extreme heat among various population subgroups (eg, age groups, gender, socioeconomic deprivation quintiles and diagnostic groups) using individual-level data from our matched cohort, applying the same model specification on each population subgroup.

#### Effect modification by green space exposure, contact with nature and air pollution (objective 4)

If we identify an association between extreme heat and mental health, we will explore the potential effect modification of green space exposure, self-reported contact with nature and each of the air pollutants (PM_2.5_, PM_10_ and NO_2_) by including measures of these exposures as an interaction factor in our models. For green space exposure, we will additionally explore whether the strength of effect modification varies for subcategories of green space, according to the seven categories of green space outlined in The London Plan.^51^

#### Confounding variables

Where suitable, models will be adjusted for factors which may confound the association between extreme heat and mental health outcomes or service use, such as sociodemographic, clinical and environmental covariates. Potential confounders will be identified a priori, through subject knowledge and literature reviews, with our causal assumptions explored with directed acyclic graphs.^60^ Within each dataset, we will assess and compare the performance and fit of competing models with model fit statistics.

#### Sensitivity analyses

We will carry out various sensitivity analyses to test the generalisability and robustness of our results. First, we will examine alternative definitions of extreme heat events (eg, temperatures above the 99th percentile, varying the minimum duration for a heatwave, or including requirements on minimum night-time temperatures^45^) to see how this impacts our study outcomes. We will fit separate models using different maximum daytime temperature thresholds (vs ≥28.0°C) to define extreme heat and compare the effect sizes and fit statistics of these models to see whether other thresholds better fit our data.

For models including lagged effects, we will refit the models by considering various lag periods and use model fit statistics to select the most parsimonious model. For the Bayesian hierarchical model, model performance will be assessed with the deviance information criterion and the widely applicable Watanabe-Akaike information criterion.

Finally, we will explore the effects of temporal differences in the datasets by limiting CRIS analyses to the shared period of 2013–2023. We will also explore ways to account for the impact of the COVID-19 pandemic, such as restricting analyses to pre-2020 data or including an indicator variable for pandemic and postpandemic observations.

#### Missing data

Data will be checked for completeness, and missing data will be examined for the mechanism of missingness. Where Urban Mind data are considered to be missing at random, we will use multiple imputation, supported by London temperature, pollution and green space data, to address missing data from skipped assessments.

#### Assess and forecast the mental health and social care burden that could be attributable to extreme heat in a vulnerable urban population (objective 5)

We will develop a mental health and economic impact assessment model to estimate the mental health and economic consequences of extreme heat events in vulnerable urban communities, and the mitigation of these impacts by different categories of urban greening and pollution-reduction policies. This model will be developed using a microsimulation approach^61^ to produce longitudinal projections of temperature-related changes in mental health.

We will first derive the effect estimates of the relationship between extreme heat events and mental health in our vulnerable groups of interest. To estimate the percentage of mental health service use that could be attributable to extreme heat, we will then calculate population attributable fractions for different exposure scenarios. The analysis will include direct healthcare costs (eg, inpatient and outpatient services), broader costs (eg, medications, social care) and mortality-related economic values (eg, life-years lost or gained).

We will then compare these effect estimates across different urban greening and pollution-reduction policy scenarios, which could act as strategies for urban heat island mitigation. We will take different approaches to ensure our findings are both scientifically informative and actionable for urban planning. For example, we will model a uniform greenness increase scenario, in which green spaces increase by 10% over the next 5 years; while unlikely to be achievable in all areas, this approach will allow us to approximate an upper bound of potential health benefits under city-wide greening. Another scenario we will consider is if mean concentrations of PM_2.5_ in our study reduced to the WHO’s recommended annualised mean limit (5.0 µg/m³), thereby providing a clear counterfactual scenario of increased greenness and reduced pollution. This microsimulation model could be extended to other urban areas to derive location-specific effect estimates of the temperature–mental health relationship. We will also develop a spatially explicit scenario in collaboration with urban policy-makers, planners and landscape architects to identify locations where new tree canopies and other urban green spaces (eg, pocket parks) are most feasible and prioritise communities that are currently underserved. This approach reflects realistic land-use opportunities and highlights the potential for targeted interventions to reduce inequities in access to green space.

### Patient and public involvement

Patient and public involvement in this study is composed of two strands: stakeholder interviews with people with lived experience and mental health professionals and a public engagement programme for London residents.

#### Stakeholder interviews (objective 6)

We will conduct six focus groups with people with lived experience of heat exposure or mental illness and healthcare professionals to codevelop a screening tool and set of recommendations with the aim to identify and support mental health service users most vulnerable to extreme heat. Participants will be recruited from the general public using purposive and snowball sampling via the King’s College London recruitment newsletter and word of mouth. The recruitment materials will particularly encourage participation from people with a history of mental illness or mental health professionals.

From the pool of interested people, the research team will select participants to ensure a variation in age, gender and ethnicity within each focus group, while also prioritising people with a history of mental illness or mental health professionals.

The focus groups will be run in three stages: responses in the first stage will be used to draft the first versions of the screening tool and set of recommendations; these drafts will then be discussed and iteratively revised in the second and third stage. Each stage will comprise two focus groups: one with people with lived experience of heat exposure or mental illness and another with healthcare professionals.

The semistructured topic guides for all three stages will be designed to answer the following research questions: (1) How does extreme heat affect the mental health of people with lived experience? (2) What are the mechanisms underpinning the impact of extreme heat on mental health? (3) What are risk and protective factors for experiencing mental health issues during extreme heat? (4) What are coping strategies people with lived experience use to prevent mental health issues during extreme heat?

The focus groups will last 1.5 hours and will be co-moderated by two members of the research team. They will be audiorecorded and transcribed verbatim. The transcripts will be analysed using thematic content analysis following Green and Thorogood,^62^ with separate analyses for the four research questions. The analyses will be primarily deductive, as the aims are predefined and some factors will be prompted in the topic guides; however, there will also be inductive elements, because new themes can arise in the discussions. Two separate coding frames will be developed for the lived experience and healthcare professional groups. The coding frameworks will be revised iteratively throughout the three stages and coded in NVivo software. The coding and theme development will be done through ongoing discussions between members of the research team.

#### Public engagement programme for London residents (objective 6)

A lack of accessible and inclusive green and blue spaces can lead to disconnection from the natural world among urban communities, reducing opportunities and confidence for outdoor recreation and mental restoration. This disconnection can negatively affect physical and mental well-being and limit engagement with environmental issues, reinforcing a cycle where urban residents run the risk of becoming further removed from the responsibilities and benefits of ecological kinship. To explore these issues, we will partner with the Bethnal Green Nature Reserve (BGNR),^63^ a grassroots organisation with a focus on the integration of urban ecology and public health, to provide a public engagement programme for London residents.

With over a decade of experience and a strong London-wide network, the BGNR provides unique natural assets—including woodlands, wetlands and food-growing gardens—alongside outdoor learning spaces and an established intergenerational public audience of London residents. The BGNR public engagement programme will be structured as an ‘alternative school’ and will integrate expert-led talks, workshops and field trips. A year-long Climate & Health talks series will feature leading voices in climate, ecology and health, providing a public platform for project partners to share research and insights with audiences across London. In parallel, weekly community-led workshops on climate change and social and ecological health will take place within the BGNR across 2026. This initiative continues BGNR’s Community Ecologies series of colearning workshops,^64^ fostering accessible grassroots knowledge exchange and skill-sharing.

The planned public engagement will take place on-site, across London and online, enabling engagement with residents across London and ensuring that it reflects the lived experiences of people represented in the CRIS and Urban Mind datasets. The programme will focus on the challenges and opportunities of engaging with urban nature in our changing climate, drawing on the lived experiences of local communities. Our goal is to instigate community-driven support structures that encourage meaningful engagement with green and blue spaces. The learnings from the programme will lead to the publication of a practical guide on how local communities can mitigate and adapt to the impacts of climate change on ecological and human health.

### Industry impact (objective 7)

An effective heat resilience strategy must integrate both short-term and long-term solutions. Interventions such as establishing dedicated cooling centres and improving public awareness around heat risks can provide immediate impact. However, longer-term solutions require consideration and redesign of our physical surroundings to improve thermal comfort. Factors such as shade, surface reflectivity, humidity and pollution can all amplify the effects of extreme heat on mental well-being.

Urban greening can cool cities and has the potential to support mental health among multiple pathways in a way that other cooling interventions do not. Landscape architects are uniquely equipped with skills to design with nature for the health and well-being of people and to foster a healthy natural environment. One of the most powerful design tools to achieve this is trees. Large canopy trees provide a multitude of long-term benefits including increased biodiversity and habitat creation, carbon sequestration, filtering airborne pollutants, shade, evapotranspiration^65^ and heat dissipation, interception and moisture retention.

Recognising the widespread benefits of trees, the Mayor of London is targeting a 10% increase in tree canopy by 2050.^24^ However, in urban areas, 20% of newly planted trees succumb to poor growing conditions.^66^ To reach maturity, trees require sufficient below-ground root zone for gaseous exchange, water flows and root growth, together with reduced compaction and enhanced stewardship skills.^67^ Evidently, we need to protect, nurture and manage the urban forest structure to achieve mature large tree canopies.

We will explore how design professionals can enable large canopies through public realm interventions to enhance human thermal comfort and mitigate the negative impacts of extreme heat on mental well-being. By incorporating results from epidemiological analyses and green space mapping, we will focus on developing feasible, scalable solutions: by understanding what makes tree canopy programmes successful in some contexts but not others, we can provide industry partners with actionable, evidence-based recommendations on where and how to invest in urban greening to yield both thermal and mental health benefits.

In practice, this exploration will involve a desktop study to understand current design guidelines and policy gaps impacting the quality and longevity of mature large canopy trees in London; cartographic analysis at a London and borough scale, supported by the results of our epidemiological analyses, to determine tree planting or retrofitting opportunities according to factors such as Transport for London’s nine street typologies, street parking, existing and proposed cycle routes, deprivation indices, existing canopy tree density, and tree species, size and age; and field observations to ground-truth the quality and conditions of priority trees identified through the cartographic analysis.

Outputs from this exploration will include maps that identify and spatialise potential locations for tree planting or retrofitting, and a technical document with illustrated planting and streetscape retrofit typologies and written guidelines, based on the nine street typologies in the Sustainable Urban Drainage Systems in London guidance.^68^ Visual outputs will be hosted on a publicly accessible web platform to engage the community, built environment practitioners, developers, local authorities, policy-makers, academics and stakeholders. The platform contents will promote best practices to mitigate the effects of extreme heat on mental well-being through planting large canopy trees and safeguarding the existing canopy in London within what is an increasingly engineered environment, to enhance resilience and secure London’s Urban Forest into the future.

### Policy impact (objective 8)

In recent years, there has been a rapid increase in extreme heatwaves and other climate impacts. In early 2022, the UK Committee on Climate Change estimated that there was only a small chance of 40°C heat in the UK before 2040, but only 6 months later London experienced such a deadly heatwave. The rapid onset of climate impacts means that the consequences of extreme heat on mental health—including in the most vulnerable communities and within urban heat islands—remain under-examined and poorly understood and are not adequately addressed by current climate-facing policy and regulation.

This is a highly dynamic area of public policy. For example, the UK Government is attempting to implement ambitious climate change net zero policies, in line with statutory legislation, while the Mayor of London works to meet the strategic target of a net zero carbon London by 2030, all the while retaining sufficient public support for these measures in a tight fiscal environment.

It will, therefore, be valuable to share findings from our research on the mental health aspects of extreme heat with policy-makers, to develop practical interventions, and potentially influence upgraded policy and regulatory frameworks. For example, there may be issues around access to and design of green space, building codes or early warning and other support systems adopted by medical practitioners, which could significantly mitigate the impact of extreme heat on mental health. There may also be international best practice that we can disseminate.

Within the project team, one of our policy advisors is the former UK Special Representative for Climate Change, with comprehensive connections across the UK policy landscape, nationally and in London. The project goals were raised at the early design stage with senior policy and thought leaders, including senior officials in the Greater London Authority (GLA); the London Climate Ready Partnership, which is the Mayor of London’s and the GLA’s business and civil society advisory group for climate resilience; and with colleagues at the London Councils, who coordinate policy and delivery across the boroughs, and are actively seeking inputs to their strategic climate resilience work in this field.

All those consulted supported the approach of the project and expressed interest in further discussion and policy-relevant findings, given its relevance to implementation of The London Plan and London Environment Strategy, which are leading policy documents shaping delivery of London’s climate and environment goals. Consultations also revealed the high potential relevance of the findings to other cities globally.

The project team will provide a range of policy briefings and material; present findings at a policy briefing for officials, business and civil society; and follow up with the above contacts and others to maximise influence on policy formulation.

## Integration

Integration across the eight project objectives and the various project partners will be fostered through monthly meetings, collaborative working with project partners and the public engagement programme.

We will hold monthly meetings in which academics, community groups, and industry and policy experts come together to review progress and plan the next steps. Project partners will present updates and findings to enable collective discussion on how individual progress can inform, and be informed by, the work of other project partners. Examples of this integration across partners and objectives are given below:

- The framing of our research questions was informed by input from our policy consultants (NB, MW) to maximise relevance for policy-makers, and by feedback from people with lived experience who have taken part in workshops run as part of the public engagement programme to ensure relevance for vulnerable populations.
- The public engagement programme for London residents is being developed with input from all project partners and will be informed by the results of the epidemiological analysis. For example, the programme has included the installation of a billboard in the BGNR to raise awareness of the mental health impacts of extreme heat, the content of which was codeveloped by participants in the stakeholder interviews. The programme will additionally include talks from industry partners to explore the intersections of ecological and human through a design lens and from research partners to discuss the impact of green space accessibility.
- The development of the industry impact strategy will be informed by the results of the epidemiological analyses, consultation with Transport for London and consultations taking place at the BGNR as part of the public engagement programme. With this approach, we aim to illustrate the results of the epidemiological analyses using specific streets as case studies, with solutions to extreme heat exposure informed by feedback from participants in the public engagement programme.

## Discussion

This longitudinal, population-based, record-linked natural experiment will provide much-needed insight into the effect of extreme heat on mental health within urban communities. It will allow us to explore the impact of extreme heat on vulnerable subgroups of society and further our understanding of whether environmental features of our urban environments—such as green space exposure and air pollution levels—modify the effect of extreme heat on mental health.

Our sophisticated approach will link EHR and smartphone data with state-of-the-art high-resolution spatiotemporal datasets of environmental factors, analysed using novel quasi-experimental designs. The use of large-sample, readily available EHR and smartphone data means that our analysis will be sufficiently powered to detect the effects of transient environmental exposures such as heat and air pollution. In addition, CRIS can facilitate linkage with external datasets, enabling the possibility of tracking patient journeys longitudinally after first contact with mental health services.

This strong linkage with external datasets also makes it possible to explore potential effect modifiers for the relationship between extreme heat and mental health. We will consider individual environmental exposures at a resolution rarely seen in existing studies, assessing air pollution and temperature exposures at a 1 km² resolution and green space exposure within a 300 m radius, in line with recommended standards for accessible green space.

A core strength of this study is the multidisciplinary and multiprofession team at its heart, composed of researchers, community organisations, industry partners and specialist policy experts. This breadth of expertise will ensure improved research design, broader participation of stakeholders, and that the knowledge and evidence generated from the study is translated into policy and practice.

Throughout the duration of the study, we will work with the public and people with lived experience. We will codevelop a screening tool with mental health professionals and lived experience experts and produce a set of recommendations, which could be embedded into policy to support mental health service users who are most vulnerable to the effects of extreme heat. We will additionally work with grassroots organisations, such as the BGNR, to build urban residents’ connection with their natural environment. Through this process of interaction and community engagement, we will produce guides for local communities on mitigating and adapting to the impacts of climate change, and work with industry to promote best practices in planting and securing the future of large canopy trees in an urban environment.

Potential limitations of this study may include problems inherent with retrospective analyses of EHR data, such as errors in coding, missing data and sampling bias; assessment of mental well-being in a self-selected cohort, potentially introducing selection bias; and problems generalising our findings to other urban settings that differ substantially in their temperature, air pollution, green space and healthcare provision levels. For example, our CRIS sample includes data for service users from six of 32 London boroughs, meaning our findings may not extend to other boroughs with distinct characteristics. Additionally, we are using daily averages of meteorological variables and air pollution as proxies for individual exposures, which may vary depending on individual behaviours, movements and residential facilities.

## Ethics

Each component of this project has been approved by the relevant ethics committee (reference RESCM-22/23-6905 for the Urban Mind dataset; LRS/DP-23/24-41409 for the codevelopment of a screening tool; 23/SC/0257 for the SLaM EHRs; and 24/EE/0178 for the NLFT EHRs).

## Dissemination plan

In addition to the publication of peer-reviewed scientific articles, our dissemination plan includes policy briefs in collaboration with the Policy Institute at King’s College London, a practical guide on fostering ecological and human resilience at the neighbourhood level, and a technical guide for planting and improving the growing conditions of large canopy trees to be published on a web platform to engage with broader interest groups.
