# Supplementary material for: A longitudinal, population-based, record-linked natural experiment on the effects of extreme heat events on mental health in urban communities: a study protocol
Source: BMJ Open. 2025 Oct 21;15(10):e099771. doi: 10.1136/bmjopen-2025-099771 (PMC12548616; doi:10.1136/bmjopen-2025-099771)
Supplement: Supplementary file 1 [file bmjopen-2025-099771.reviewer_comments.pdf]

PEER REVIEW HISTORY

BMJ Open publishes all reviews undertaken for accepted manuscripts. Reviewers are asked to complete a checklist review form and are provided with free text boxes to elaborate on their assessment. These free text comments are reproduced below.

ARTICLE DETAILS

Title (Provisional)

A study protocol for a longitudinal, population-based, record-linked natural experiment on the effects of extreme heat events on mental health in urban communities

Authors

Chukwusa, Emeka; Vivaldi, Giulia; Baecker, Lea; Bowerman, Emily; Bridge, Nick; Davidson, Neil; de la Cruz, Arturo; Gasparrini, Antonio; Gibbons, Johanna; Guerry, Anne; Hammoud, Ryan; Li, Yingjie; Lu, Yougeng; Mandle, Lisa; Osborn, David; Rydstrom, Anders; Smythe, Michael; Stewart, Robert; Tognin, Stefania; White, Matthew; Yang, Justin; Mechelli , Andrea; Bakolis, Ioannis

---

VERSION 1 - REVIEW

**Reviewer**	**1**
**Name**	**Lykins, Amy**
**Affiliation**	**UNE**
**Date**	**17-Apr-2025**
**COI**	**None**

This protocol describes a very comprehensive assessment of the effects of extreme heat on mental health in residents of various locations in the greater London area. The team is diverse and multi-disciplinary, lending credibility to the design and (later) implementation of data collection and analysis. The writing is clear and overall, excellent. This seems to be a very well thought out program of research and I look forward to reading its results. My comments are quite minor.

1. It looks like some of your text on pg. 7 is unintentionally small ("Two recent systematic reviews…)

2. Are you able to provide a bit more information on the socioeconomic status of your target region? For readers not very familiar with the SES spread of London as a whole, it would be helpful to contextualise this a bit more in text, especially given the known relationships between mental health and SES.

3. In consideration of consent for use of data from participants 3 – 17 years old, would their caregiver have given permission for the data to be used for research purposes? Or, if this is not an issue for healthcare database usage in the UK, it would be worth making that clearer.

4. Why are the longitudinal data being collected on residents of South London, but the participatory programme participants being recruited from East London?

5. What is a ‘lived experience expert?’

6. While I am very supportive of developing tree canopy in urban areas—for a whole host of reasons—I am curious as to why this is the only cooling intervention you’re examining, particularly since large canopy-type trees can take a while to grow? In the meantime, are there other options that could be included (at least in policy recommendations) such as dedicated cooling centres? Outreach to vulnerable people (e.g., the elderly) and/or those without air conditioning to encourage community cohesion and shared responsibility for neighbourhood wellbeing?

Reviewer 2

Name Spangler, Keith

Affiliation Boston University School of Public Health, Department of Environmental Health

Date 05-Aug-2025

COI None

This manuscript is a protocol describing a planned research project assessing the mental health impacts of extreme heat in London. The project will draw upon comprehensive electronic health records, as well as from self-reported data from a mobile app. The planned activities also include collaborations with urban planners and community partners to develop mitigation solutions and education. The protocol is very well written, innovative, and very likely to produce interesting and important findings with practical applications.

The epidemiological and statistical methods are excellent and well-documented. I have just a few minor comments on these aspects, which I’ve listed below under "Minor Comments."

However, the objectives relating to the qualitative research methods, partnerships with community groups, and policymaker engagement are under-developed and need additional details. These comments are listed below under "Major Comments."

____

Minor Comments

____

[1] A bit more description of exactly what Urban Mind is would be helpful (e.g., why was it created, who uses it, what is the objective of the project, etc.). These details are in the cited publications, but the basics should be here as well. Please also ensure that you define what "EMA" stands for.

[2] In the description of SLaM and NLFT: Please clarify what types of data are included in CRIS (e.g., ICD codes, dates of treatment, etc.).

[3] Please provide a general description of the InVEST Urban Cooling Model.

[4] It would be useful to clarify how you conceptualize "confounders": will you use a statistical definition, an a priori determination (such as through the use of directed acyclic graphs [DAGs]), or a combination of the two?

[5] COVID-19 in the "Sensitivity Analyses" section – it may be worthwhile to consider expanding on this into a standalone analysis. It's perhaps outside of scope, but I think you have a unique opportunity given the breadth of your datasets to interrogate things like the interplay between lack of AC, lockdown, and mental health.

[6] Objective 5 (page 21) - please clarify whether you plan to assess the impact of changes in urban green space in a spatially explicit way (i.e., considering specifically where it is plausible for London to have new parks or plant trees) for each unit of analysis vs. a generic 10% increase in greenness exposure across all observations. In other words, suppose you are looking at the health benefits that would come from a scenario in which London increases its greenness by a city-wide average of 10%. Will you model this as a 10% increased greenness for all individuals, or will you have person-specific greenness changes based on where it is more likely that the greenness increases could be achieved? I assume the latter, but clarifying that would benefit the protocol. This comment is related to some of my major comments below, particularly the role that the urban planners will play in the project.

Major Comments

---

[1] The primary weakness of the protocol in its current state is the lack of clarity on how the community groups, industry partners, and policymakers will be engaged in the research project. Specifically, it's unclear whether and to what extent there is feedback between the different aspects of the project. For example, will the researchers develop the research questions with policymakers at the outset, in order to ensure that the data generated is of use to them in their decision-making? Will the landscape urban planners take into account the health-impact assessments from the epidemiologists before and during their analyses (i.e., will they be looking to plant trees and open parks in a way that optimizes health benefits)? Adding a section that clearly delineates how the various teams on the project will work together to ensure that the research products are useful would greatly improve the protocol. I give a few specific comments about this below.

[2] Stakeholder interviews (Objective 6) - please provide more information about your qualitative methods, including more details on recruitment and participant selection, how you plan to systematically analyze the qualitative data, and other aspects of the qualitative research methodology.

[3] "Participatory programme for East London residents" section: This section is unclear without more context. Please elaborate on how the program will be meaningfully integrated with the research, clarify what the program is and who it serves, and add citations throughout where applicable.

[4] "Industry Impact" section (Objective 7): Similar to above, this objective feels disconnected from the rest of the research plan. Please elaborate on how it will be meaningfully integrated into the research.

[5] Objective 8 is under-developed. Which types of policymakers do you plan to engage? How specifically do you plan to engage with them? Will you communicate with them prior to beginning the work to learn what type of information is useful to them?

VERSION 1 - AUTHOR RESPONSE

Reviewer 1

This protocol describes a very comprehensive assessment of the effects of extreme heat on mental health in residents of various locations in the greater London area. The team is diverse and multi-disciplinary, lending credibility to the design and (later) implementation of data collection and analysis. The writing is clear and overall, excellent. This seems to be a very well thought out program of research and I look forward to reading its results. My comments are quite minor.

1. It looks like some of your text on pg. 7 is unintentionally small ("Two recent systematic reviews...)

Response:

Thank you for flagging; we have corrected this formatting error.

---

2. Are you able to provide a bit more information on the socioeconomic status of your target region? For readers not very familiar with the SES spread of London as a whole, it would be helpful to contextualise this a bit more in text, especially given the known relationships between mental health and SES.

Response:

We have added a little more detail on the socioeconomic positioning of the six boroughs included in our CRIS analyses, with comparisons to the London and England averages, in the 'SLaM and NLFT CRIS' section of the Methods:

"After housing costs, average poverty levels in the four [SLaM] boroughs are largely similar to the London average, and significantly higher in Lewisham than the England average.[1] However, inequality is high, with both Croydon and Southwark home to areas in the highest and lowest decile of the Index of Multiple Deprivation (IMD)."

"The two boroughs [of Camden and Islington] differ substantially in their socioeconomic status, with Camden having the highest poverty rate after housing costs in London, at twice the level of Islington, whose rate is significantly lower than the London average.[1] As with the SLaM boroughs, inequality in Camden and Islington is high."

1. Trust for London. Poverty rates by London borough (2023/24). https://trustforlondon.org.uk/data/poverty-borough/ (accessed Aug 13, 2025).

---

3. In consideration of consent for use of data from participants 3 – 17 years old, would their caregiver have given permission for the data to be used for research purposes? Or, if this is not an issue for healthcare database usage in the UK, it would be worth making that clearer.

Response:

CRIS operates an opt-out policy, meaning that patients and their caregivers have the right to opt out of their data being used in CRIS by contacting their mental health worker or the CRIS administrative team. We have added the following line to the 'SLaM and NLFT CRIS' section to clarify:

"Patients in either NHS Foundation Trust can opt out of their data being included in CRIS by contacting their mental health workers or the CRIS administrative team."

---

4. Why are the longitudinal data being collected on residents of South London, but the participatory programme participants being recruited from East London?

Response:

This is largely down to existing partnerships and connections held by the collaborators on the project, but we are aiming for elements of the public engagement programme to extend across London.

As the core research team is based at King's College London, we are taking advantage of existing partnerships with the South London & Maudsley NHS Foundation Trust. We are also leveraging data from the (formerly) Camden & Islington NHS Foundation Trust, meaning that our epidemiological analyses cover boroughs in both south and north London.

Our project partner, the Bethnal Green Nature Reserve (BGNR) in east London, has established infrastructure (including staff, established audience, woodlands, a food-growing garden, an event space, a medicinal garden, wetlands, a public engagement programme, and a forest school) that is well suited to the participatory programme. While most activities at the BGNR are expected to be attended primarily by local residents and their existing London-wide community, all events will be open to participants from across Greater London. This ensures that our planned public engagement efforts will reach a London-wide audience. We have revised the text around the participatory programme to reflect this.

Furthermore, a successful implementation of the participatory programme in the BGNR would lead to recommendations from our project team that similar programmes be rolled out in other areas, including the boroughs covered by our epidemiological analyses.

---

5. What is a 'lived experience expert?'

Response:

We primarily mean people who have lived experience of mental illness, but also people who have lived experience of heat exposure. We have rephrased 'lived experience expert' to 'people with lived experience' throughout to clarify.

---

6. While I am very supportive of developing tree canopy in urban areas—for a whole host of reasons—I am curious as to why this is the only cooling intervention you're examining, particularly since large canopy-type trees can take a while to grow? In the meantime, are there other options that could be included (at least in policy recommendations) such as dedicated cooling centres? Outreach to vulnerable people (e.g., the elderly) and/or those without air conditioning to encourage community cohesion and shared responsibility for neighbourhood wellbeing?

Response:

Thank you for this comment. We agree that a mix of short-term and long-term interventions is essential for an effective heat resilience strategy, and we strongly advocate for an integrated approach in future policy recommendations and will do so in future publications linked to this project.

However, our project was specifically developed to examine the mental health impacts of extreme heat and how these might be mitigated by urban nature. One reason for focusing on urban nature is that it may not only cool cities but also bring direct mental health benefits. In other words, it supports mental health through multiple pathways in a way that other cooling interventions (e.g., air conditioning) do not. Additionally, urban nature—and urban tree canopies in particular—offer one of the most sustainable, long-term solutions to mitigating the urban heat

island effect, while also delivering a host of co-benefits such as improved air quality, stormwater management, and biodiversity.

Given the focus of our project on urban nature, we have partnered with J&L Gibbons, who are experts in the effective creation and support of tree canopy, hence the focus on this intervention within our project. As part of our project, they are developing a strategy for making urban tree planting more effective in the long term, given the threat to London's existing canopy from climate change.

Additionally, as part of the project, we are running a London-wide survey on people's experiences of extreme heat and mental health. As part of this survey, we will compare public perception of the mental health benefits of tree canopy against other cooling solutions, such as dedicated cooling centres. Alternative cooling solutions will be included this in the survey report.

We have adjusted the introduction to our Industry impact section to provide some context for our focus on tree canopy support and development:

"An effective heat resilience strategy must integrate both short-term and long-term solutions. Interventions such as establishing dedicated cooling centres and improving public awareness around heat risks can provide immediate impact. However, longer term solutions require consideration and redesign of our physical surroundings to improve thermal comfort. Factors such as shade, surface reflectivity, humidity, and pollution can all amplify the effects of extreme heat on mental wellbeing.

Urban greening can cool cities, and has the potential to support mental health among multiple pathways in a way that other cooling interventions do not. Landscape architects are uniquely equipped with skills to design with nature for the health and wellbeing of people and to foster a healthy natural environment. One of the most powerful design tools to achieve this are trees…."

Reviewer 2

This manuscript is a protocol describing a planned research project assessing the mental health impacts of extreme heat in London. The project will draw upon comprehensive electronic health records, as well as from self-reported data from a mobile app. The planned activities also include collaborations with urban planners and community partners to develop mitigation solutions and education. The protocol is very well written, innovative, and very likely to produce interesting and important findings with practical applications.

The epidemiological and statistical methods are excellent and well-documented. I have just a few minor comments on these aspects, which I've listed below under "Minor Comments."

However, the objectives relating to the qualitative research methods, partnerships with community groups, and policymaker engagement are under-developed and need additional details. These comments are listed below under "Major Comments."

Minor Comments

---

[1] A bit more description of exactly what Urban Mind is would be helpful (e.g., why was it created, who uses it, what is the objective of the project, etc.). These details are in the cited publications, but the basics should be here as well. Please also ensure that you define what "EMA" stands for.

Response:

We have added some further descriptions of the Urban Mind study to the 'Urban Mind' section of the Methods. Please note that EMA (ecological momentary assessment) is defined at first mention in the 'Study design' section.

"Urban Mind is a smartphone-based citizen science project on the impacts of the urban environment on mental health (http://www.urbanmind.info). This EMA app was developed as part of the Urban Mind research project— a collaboration between King's College London, landscape architects J&L Gibbons, and arts foundation Nomad Projects—which aims to inform future urban planning and social policy to improve design and health in urban environments. By relying on the repeated sampling approach of EMAs, the app is designed to provide insight into dynamic changes in mental states while maximising ecological validity by collecting data in real-world environments.[1] The app has been in use across various research projects since 2015,[1] with the current version launched in May 2018. Participation in the project is self-selected and anonymous, and has been encouraged through the project website and social media platforms.[1] The dataset includes more than 56,000 assessments from over 5600 people globally, with collection ongoing. Approximately a third of the Urban Mind cohort have a diagnosis of mental illness. The Urban Mind app is available for both Apple iPhone and Android devices and is free to use. Additional details about the Urban Mind project and the app can be found in previous publications.[1,2]"

1. Bakolis I, Hammoud R, Smythe M, et al. Urban Mind: Using Smartphone Technologies to Investigate the Impact of Nature on Mental Well-Being in Real Time. *BioScience* 2018; **68**(2): 134-45.
2. Hammoud R, Tognin S, Smythe M, et al. Smartphone-based ecological momentary assessment reveals an incremental association between natural diversity and mental wellbeing. *Scientific Reports* 2024; **14**(1): 7051.

---

[2] In the description of SLaM and NLFT: Please clarify what types of data are included in CRIS (e.g., ICD codes, dates of treatment, etc.).

Response:

We have included the following information in the revised manuscript.

"CRIS contains a comprehensive record of all clinical information recorded throughout patients' journeys within Trust services. This includes patients' demographic data (e.g., gender, self-assigned ethnicity), details of referrals and transfers, clinical assessments, care plans and medication, and clinical activity and reviews, from both structured and unstructured free text sources (e.g., routine case notes, correspondence, and medication notes).[1,2] CRIS also includes

details of mental health outcomes such as the Mini-Mental State Examination and Health of the Nation Outcome Scales (HoNOS).[3]"

1. Perera G, Broadbent M, Callard F, et al. Cohort profile of the South London and Maudsley NHS Foundation Trust Biomedical Research Centre (SLaM BRC) Case Register: current status and recent enhancement of an Electronic Mental Health Record-derived data resource. *BMJ Open* 2016; **6**(3): e008721.
2. Werbeloff N, Osborn DPJ, Patel R, et al. The Camden & Islington Research Database: Using electronic mental health records for research. *PLoS One* 2018; **13**(1): e0190703.
3. Stewart R, Soremekun M, Perera G, et al. The South London and Maudsley NHS Foundation Trust Biomedical Research Centre (SLAM BRC) case register: development and descriptive data. *BMC Psychiatry* 2009; **9**: 51.

---

[3] Please provide a general description of the InVEST Urban Cooling Model.

Response:

We have added the following text to the manuscript:

"The link between green space exposure and heat assessment will then be adapted to run the InVEST Urban Cooling Model (UCM), developed by the Natural Capital Project at Stanford University. This model is part of a widely used toolkit for assessing ecosystem services.[1] The UCM estimates heat mitigation by incorporating the effects of shade, evapotranspiration, albedo, and proximity to cooling features such as parks. It further translates land-use and land-cover patterns or scenarios into projected changes in urban heat island intensity, enabling evaluation of how green infrastructure contributes to reducing heat stress and supporting human health. This model will be applied across various climate and land-use scenarios to evaluate the benefits of green space on mental health outcomes."

1. Natural Capital Project. InVEST 3.16.2. Stanford University, University of Minnesota, Chinese Academy of Sciences, The Nature Conservancy, World Wildlife Fund, Stockholm Resilience Centre, and the Royal Swedish Academy of Sciences, 2025.

---

[4] It would be useful to clarify how you conceptualize "confounders": will you use a statistical definition, an a priori determination (such as through the use of directed acyclic graphs [DAGs]), or a combination of the two?

Response:

We have added the following line to our 'Confounding variables' section to clarify:

"Potential confounders will be identified a priori, through subject knowledge and literature reviews, with our causal assumptions explored with directed acyclic graphs.[1]"

1 Greenland S, Pearl J, Robins JM. Causal Diagrams for Epidemiologic Research. *Epidemiology* 1999; **10**(1).

---

[5] COVID-19 in the "Sensitivity Analyses" section – it may be worthwhile to consider expanding on this into a standalone analysis. It's perhaps outside of scope, but I think you have a unique

opportunity given the breadth of your datasets to interrogate things like the interplay between lack of AC, lockdown, and mental health.

Response:

Thank you for this suggestion. We agree that exploring the interplay between housing quality, heat, and mental health during the COVID-19 lockdowns is important, particularly given the increased time that the population spent at home and the likely reduction in green space exposure. Unfortunately, this is outside of the scope of the present study, owing to time and data constraints. In particular, we lack data on housing type, quality, and indoor cooling, which we feel would be essential data to interrogate this topic thoroughly. In a future project, we will consider expanding the analysis, perhaps as a follow-on study to explore these research questions with better data linkage to housing types and cooling.

---

[6] Objective 5 (page 21) - please clarify whether you plan to assess the impact of changes in urban green space in a spatially explicit way (i.e., considering specifically where it is plausible for London to have new parks or plant trees) for each unit of analysis vs. a generic 10% increase in greenness exposure across all observations. In other words, suppose you are looking at the health benefits that would come from a scenario in which London increases its greenness by a city-wide average of 10%. Will you model this as a 10% increased greenness for all individuals, or will you have person-specific greenness changes based on where it is more likely that the greenness increases could be achieved? I assume the latter, but clarifying that would benefit the protocol. This comment is related to some of my major comments below, particularly the role that the urban planners will play in the project.

Response:

We plan to use different approaches and scenarios to provide a comprehensive understanding of the potential health benefits of expanding urban green space. We have modified the text around Objective 5 as follows to clarify:

"We will then compare these effect estimates across different urban greening and pollution-reduction policy scenarios, which could act as strategies for urban heat island mitigation. We will take different approaches to ensure our findings are both scientifically informative and actionable for urban planning. For example, we will model a uniform greenness increase scenario, in which green spaces increase by 10% over the next 5 years; while unlikely to be achievable in all areas, this approach will allow us to approximate an upper bound of potential health benefits under city-wide greening. Another scenario we will consider is if mean concentrations of $PM_{2.5}$ in our study reduced to the World Health Organization's recommended annualised mean limit (5.0 μg/m^3), thereby providing a clear counterfactual scenario of increased greenness and reduced pollution. This microsimulation model could be extended to other urban areas to derive location-specific effect estimates of the temperature–mental health relationship. We will also develop a spatially explicit scenario in collaboration with urban policymakers, planners, and landscape architects to identify locations where new tree canopies and other urban green spaces (e.g., pocket parks) are most feasible and prioritising

communities that are currently underserved. This approach reflects realistic land-use opportunities and highlights the potential for targeted interventions to reduce inequities in access to green space."

Major Comments

[1] The primary weakness of the protocol in its current state is the lack of clarity on how the community groups, industry partners, and policymakers will be engaged in the research project. Specifically, it's unclear whether and to what extent there is feedback between the different aspects of the project. For example, will the researchers develop the research questions with policymakers at the outset, in order to ensure that the data generated is of use to them in their decision-making? Will the landscape urban planners take into account the health-impact assessments from the epidemiologists before and during their analyses (i.e., will they be looking to plant trees and open parks in a way that optimizes health benefits)? Adding a section that clearly delineates how the various teams on the project will work together to ensure that the research products are useful would greatly improve the protocol. I give a few specific comments about this below.

Response:

We acknowledge that integration among the different components of the project and the different types of expertise is important but also challenging. Truly inter-disciplinary and inter-sector research is rare, and we are still learning how to best achieve it.

In order to bring different elements of the project together, we are holding monthly meetings in which academics, industry partners, community groups (i.e., representatives of the Bethnal Green Nature Reserve Trust), and policy experts come together to review progress and plan the next steps. In a typical meeting, a project partner presents their work to date, followed by a collective discussion on how this can inform, and be informed by, the work of the other partners. This iterative process has led to several instances of cross-pollination of ideas.

To make this clearer in the manuscript, we have added the following Integration section before the Discussion, in which we outline our approach to integrating the work and output around each objective, with examples of how this has been achieved to date. We have additionally revised some of the other objectives sections (as detailed in our other responses) to better highlight how different project partners and objectives are linked.

"**Integration**

Integration across the eight project objectives and the various project partners will be fostered through monthly meetings, collaborative working with project partners, and the public engagement programme.

We will hold monthly meetings in which academics, community groups, and industry and policy experts come together to review progress and plan the next steps. Project partners will present

updates and findings to enable collective discussion on how individual progress can inform, and be informed by, the work of other project partners. Examples of this integration across partners and objectives are given below:

- The framing of our research questions was informed by input from our policy consultants (NB, MW) to maximise relevance for policy makers, and by feedback from people with lived experience who have taken part in workshops run as part of the public engagement programme to ensure relevance for vulnerable populations.
- The public engagement programme for London residents is being developed with input from all project partners and will be informed by the results of the epidemiological analyses. For example, the programme has included the installation of a billboard in the BGNR to raise awareness of the mental health impacts of extreme heat, the content of which was co-developed by participants in the stakeholder interviews. The programme will additionally include talks from industry partners to explore the intersections of ecological and human through a design lens and from research partners to discuss the impact of green space accessibility.
- The development of the industry impact strategy will be informed by the results of the epidemiological analyses, consultation with Transport for London, and consultations taking place at the BGNR as part of the public engagement programme. With this approach, we aim to illustrate the results of the epidemiological analyses using specific streets as case studies, with solutions to extreme heat exposure informed by feedback from participants in the public engagement programme."

---

[2] Stakeholder interviews (Objective 6) - please provide more information about your qualitative methods, including more details on recruitment and participant selection, how you plan to systematically analyze the qualitative data, and other aspects of the qualitative research methodology.

Response:

Please see our adjustments to the section 'Stakeholder interviews (Objective 6)' below:

Stakeholder interviews (Objective 6)

We will conduct six focus groups with people with lived experience of heat exposure or mental illness and healthcare professionals to co-develop a screening tool and set of recommendations with the aim to identify and support mental health service users most vulnerable to extreme heat. Participants will be recruited from the general public using purposive and snowball sampling via the King's College London recruitment newsletter and word of mouth. The recruitment materials will particularly encourage participation from people with a history of mental illness or mental health professionals.

From the pool of interested people, the research team will select participants to ensure a variation in age, gender, and ethnicity within each focus group, while also prioritising people with a history of mental illness or mental health professionals.

The focus groups will be run in three stages: responses in the first stage will be used to draft the first versions of the screening tool and set of recommendations; these drafts will then be discussed and iteratively revised in the second and third stage. Each stage will comprise two focus groups: one with people with lived experience of heat exposure or mental illness and another with healthcare professionals.

The semi-structured topic guides for all three stages will be designed to answer the following research questions: (1) How does extreme heat affect the mental health of people with lived experience? (2) What are the mechanisms underpinning the impact of extreme heat on mental health? (3) What are risk and protective factors for experiencing mental health issues during extreme heat? (4) What are coping strategies people with lived experience use to prevent mental health issues during extreme heat?

The focus groups will last 1.5h and will be co-moderated by two members of the research team. They will be audio-recorded and transcribed verbatim. The transcripts will be analysed using thematic content analysis following Green and Thorogood,[1] with separate analyses for the four research questions. The analyses will be primarily deductive, as the aims are predefined and some factors will be prompted in the topic guides; however, there will also be inductive elements, because new themes can arise in the discussions. Two separate coding frames will be developed for the lived experience and healthcare professional groups. The coding frameworks will be revised iteratively throughout the three stages and coded in NVivo software. The coding and theme development will be done through ongoing discussions between members of the research team.

1. Green J, Thorogood N. Qualitative methods for health research. 4th ed. London: Sage; 2018.

---

[3] "Participatory programme for East London residents" section: This section is unclear without more context. Please elaborate on how the program will be meaningfully integrated with the research, clarify what the program is and who it serves, and add citations throughout where applicable.

Response:

We have added further details as follows, including references to the Bethnal Green Nature Reserve and its relevant programmes.

"Public engagement programme for London residents (Objective 6)

A lack of accessible and inclusive green and blue spaces can lead to disconnection from the natural world among urban communities, reducing opportunities and confidence for outdoor recreation and mental restoration. This disconnection can negatively affect physical and mental wellbeing and limit engagement with environmental issues, reinforcing a cycle where urban residents run the risk of becoming further removed from the responsibilities and benefits of ecological kinship. To explore these issues, we will partner with the Bethnal Green Nature

Reserve (BGNR),[1] a grassroot organisation with a focus on the integration of urban ecology and public health, to provide a public engagement programme for London residents.

With over a decade of experience and a strong London-wide network, the BGNR provides unique natural assets—including woodlands, wetlands, and food-growing gardens—alongside outdoor learning spaces and an established intergenerational public audience of London residents. The BGNR public engagement programme will be structured as an 'alternative school', and will integrate expert-led talks, workshops, and field trips. A year-long Climate & Health talks series will feature leading voices in climate, ecology, and health, providing a public platform for project partners to share research and insights with audiences across London. In parallel, weekly community-led workshops on climate change and social and ecological health will take place within the BGNR across 2026. This initiative continues BGNR's Community Ecologies series of co-learning workshops,[2] fostering accessible grassroots knowledge exchange and skill-sharing.

The planned public engagement will take place on-site, across London, and online, enabling engagement with residents across London and ensuring that it reflects the lived experiences of people represented in the CRIS and UM datasets. The programme will focus on the challenges and opportunities of engaging with urban nature in our changing climate, drawing on the lived experiences of local communities. Our goal is to instigate community-driven support structures that encourage meaningful engagement with green and blue spaces. The learnings from the programme will lead to the publication of a practical guide on how local communities can mitigate and adapt to the impacts of climate change on ecological and human health.

1. Bethnal Green Nature Reserve. https://www.bethnalgreennaturereserve.org/ (accessed Aug 29, 2025).
2. Community Ecologies Programme 2025. https://bethnalgreennaturereserve.org/projects/community-ecologies (accessed Aug 29, 2025).

---

[4] "Industry Impact" section (Objective 7): Similar to above, this objective feels disconnected from the rest of the research plan. Please elaborate on how it will be meaningfully integrated into the research.

Response:

As outlined in response to Reviewer 1 (comment 6), we have modified the introduction to the Industry impact section to provide more context for our focus on tree canopies as a cooling intervention. Furthermore, we have mentioned explicitly in the new 'Integration' section how the work by our industry project partners will feed into the participatory programme, as well as clarifying how the modelling team will work with our industry partners to identify locations for new tree canopies. Finally, we have clarified within the Industry impact section how other strands of the project can help with the development of feasible, scalable industry solutions (as shown below). We hope these various edits better reflect the integration of our industry partners' work with the wider project.

"We will explore how design professionals can enable large canopies through public realm interventions to enhance human thermal comfort and mitigate the negative impacts of extreme heat on mental wellbeing. By incorporating results from epidemiological analyses and green space mapping, we will focus on developing feasible, scalable solutions: by understanding what makes tree canopy programs successful in some contexts but not others, we can provide industry partners with actionable, evidence-based recommendations on where and how to invest in urban greening to yield both thermal and mental health benefits.

In practice, this exploration will involve a desktop study to understand current design guidelines and policy gaps impacting the quality and longevity of mature large canopy trees in London; cartographic analysis at a London and borough scale, supported by the results of our epidemiological analyses, to determine tree planting or retrofitting opportunities according to factors such as Transport for London's nine street typologies, street parking, existing and proposed cycle routes, deprivation indices, existing canopy tree density, tree species, size, and age; and field observations to ground-truth the quality and conditions of priority trees identified through the cartographic analysis."

---

[5] Objective 8 is under-developed. Which types of policymakers do you plan to engage? How specifically do you plan to engage with them? Will you communicate with them prior to beginning the work to learn what type of information is useful to them?

Response: *Thank you for this comment. We have added the following text to Objective 8 to elaborate:*

"Within the project team, one of our policy advisors is the former UK Special Representative for Climate Change with comprehensive connections across the UK policy landscape, nationally, and in London. The project goals were raised at the early design stage with senior policy and thought leaders, including senior officials in the Greater London Authority (GLA); the London Climate Ready Partnership, which is the Mayor of London's and the GLA's business and civil society advisory group for climate resilience; and with colleagues at the London Councils, who coordinate policy and delivery across the boroughs, and are actively seeking inputs to their strategic climate resilience work in this field.

All those consulted supported the approach of the project and expressed interest in further discussion and policy relevant findings, given its relevance to implementation of The London Plan & London Environment Strategy, which are leading policy documents shaping delivery of London's climate and environment goals. Consultations also revealed the high potential relevance of the findings to other cities globally.

The project team will provide a range of policy briefings and material; present findings at a policy briefing for officials, business, and civil society; and follow up with the above contacts and others to maximise influence on policy formulation."
